# Prioritization of risk genes in multiple sclerosis by a refined Bayesian framework followed by tissue-specificity and cell type feature assessment

**DOI:** 10.1186/s12864-022-08580-y

**Published:** 2022-05-11

**Authors:** Andi Liu, Astrid M. Manuel, Yulin Dai, Zhongming Zhao

**Affiliations:** 1 Department of Epidemiology, School of Public Health, Human Genetics and Environmental Sciences, The University of Texas Health Science Center at Houston, Houston, TX 77030 USA; 2 Center for Precision Health, School of Biomedical Informatics, The University of Texas Health Science Center at Houston, Houston, TX 77030 USA; 3 Human Genetics Center, School of Public Health, The University of Texas Health Science Center at Houston, Houston, TX 77030 USA

**Keywords:** Multi-omics, Bayesian framework, Multiple sclerosis, Two-sample Mendelian randomization, Single-cell RNA-sequencing

## Abstract

**Background:**

Multiple sclerosis (MS) is a debilitating immune-mediated disease of the central nervous system that affects over 2 million people worldwide, resulting in a heavy burden to families and entire communities. Understanding the genetic basis underlying MS could help decipher the pathogenesis and shed light on MS treatment. We refined a recently developed Bayesian framework, Integrative Risk Gene Selector (iRIGS), to prioritize risk genes associated with MS by integrating the summary statistics from the largest GWAS to date (n = 115,803), various genomic features, and gene–gene closeness.

**Results:**

We identified 163 MS-associated prioritized risk genes (MS-PRGenes) through the Bayesian framework. We replicated 35 MS-PRGenes through two-sample Mendelian randomization (2SMR) approach by integrating data from GWAS and Genotype-Tissue Expression (GTEx) expression quantitative trait loci (eQTL) of 19 tissues. We demonstrated that MS-PRGenes had more substantial deleterious effects and disease risk. Moreover, single-cell enrichment analysis indicated MS-PRGenes were more enriched in activated macrophages and microglia macrophages than non-activated ones in control samples. Biological and drug enrichment analyses highlighted inflammatory signaling pathways.

**Conclusions:**

In summary, we predicted and validated a high-confidence MS risk gene set from diverse genomic, epigenomic, eQTL, single-cell, and drug data. The MS-PRGenes could further serve as a benchmark of MS GWAS risk genes for future validation or genetic studies.

**Supplementary Information:**

The online version contains supplementary material available at 10.1186/s12864-022-08580-y.

## Background

Multiple sclerosis (MS) is an immune-mediated disease of the central nervous system characterized by the dissemination of lesions in space and time with demyelination and inflammation. MS affects over 2 million people worldwide, with over 75% of MS patients being women, which imposes a heavy burden on patients, families, and the public health system [1]. Currently, there is no treatment to stop or reverse the pathogenesis of MS. Moreover, the etiology of MS is poorly understood, as it involves both genetic and environmental factors.

Epidemiologic and genetic studies have identified the critical role of genetic factors underlying MS during the recent decades [2–5]. The completion of the human reference genome and newly developed genotyping technologies have made it possible to conduct large-scale population-based association studies between cases and controls [6]. The most recent genome-wide association studies (GWAS) of 47,429 MS cases and 68,374 control subjects have reported 200 autosomal susceptibility variants outside the major histocompatibility complex (MHC) [7]. However, identifying the corresponding MS-associated risk genes is still a popular area of research, given that true risk genes may be located at a far distance from the susceptibility variants. Thus, there is still a need to prioritize the genes and functions linked to the genetic variants depicted by the MS GWAS.

Although there is a genetic component to MS onset, monozygotic twins are often discordant for MS [8]. Furthermore, there are different subtypes of MS, and the disease course may vary significantly among individuals with MS. This indicates that there is also an environmental component to MS etiology. During the past two decades, epigenetic studies have made tremendous progress in expanding our understanding of environmental factors in MS. DNA methylation is the most studied epigenetic mechanism, which plays an important role in gene expression regulation without altering DNA sequence [9]. Huynh et al. have reported that changes in DNA methylation were associated with gene expression, which affected oligodendrocyte susceptibility, and led to damage among brain samples of MS cases [8].

Considering that a large proportion of GWAS loci are in the noncoding regions of the genome, the strategy of choosing the closest gene to each index single nucleotide polymorphism (SNP) might not explain the complicated regulatory mechanisms well [10, 11]. This is because the 'real' risk gene may locate at a far distance or on different chromosomes [12]. Therefore, explicit methods that could leverage the accumulating genomic and epigenomic evidence have been developed for fine mapping these genetic risk loci in complex diseases [13–16]. However, to our knowledge, there have been few studies integrating multi-omics data [17–19] to predict the risk genes for MS.

To fill the gap, the current study aims to predict the MS-associated risk genes in the most recent GWAS of 115,803 individuals [7]. Specifically, we extended a recently developed Bayesian framework, Integrative Risk Gene Selector (iRIGS) [14], to incorporate genomic features and multi-omics information in network space to identify MS-associated prioritized risk genes. We name them as MS-PRGenes. We subsequently conducted two-sample Mendelian randomization (2SMR) analyses to validate the MS-PRGenes in 19 tissues [20]. Furthermore, we investigated their genetic features and conducted single-cell enrichment analysis in both case and control samples to explore the cell-specific functions [21, 22]. Lastly, we performed pathway and drug signature enrichment analyses of the MS-PRGenes to better understand the function of these prioritized genes and explore the potential drug repositioning strategies for MS.

## Results

### Integrative Risk Gene Selector (iRIGS)

**Fig. 1:**
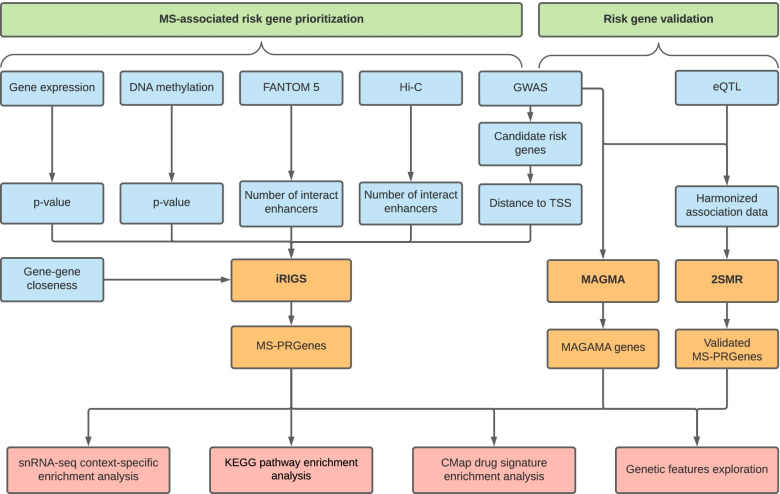
Workflow of the study. Multi-omics data input for MS-associated risk gene prioritization and validation are labeled in blue. Primary analyses of MS-associated risk gene prioritization and validation are labeled in orange. Analyses on MS-PRGenes and validation gene sets are labeled in red

**Fig. 2:**
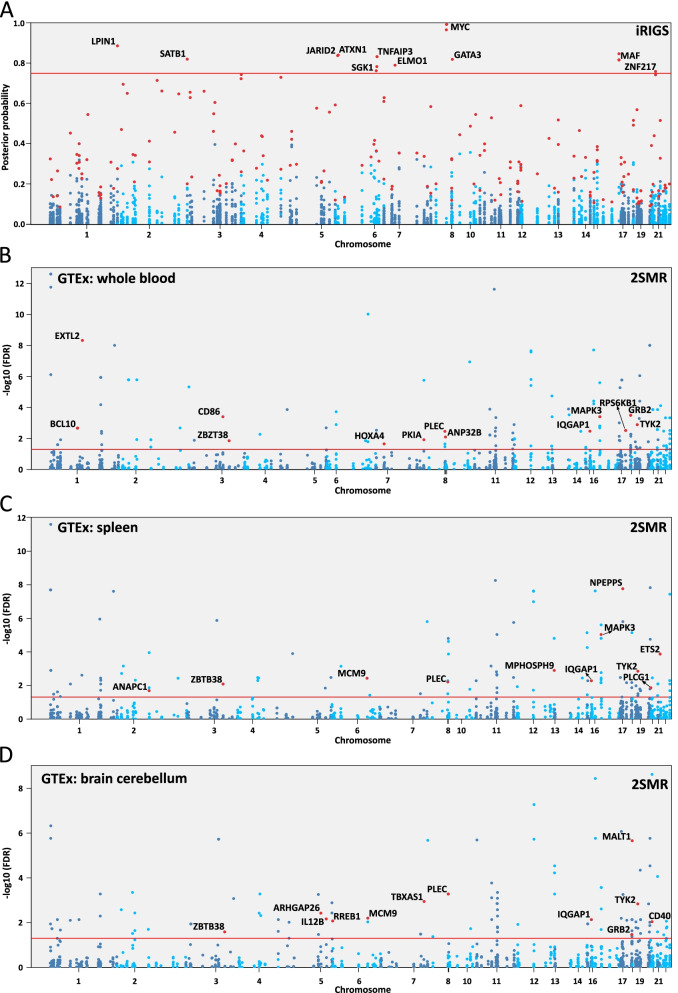
Manhattan plots of results of iRIGS and 2SMR. **A** Manhattan plot of posterior probabilities of candidate genes from iRIGS. Highlighted genes are candidate risk genes with the highest posterior probability at each index SNP. Genes are labeled with high posterior probability (> 0.75). (**B-D**) Manhattan plots of 2SMR analyses on whole blood, spleen, and brain cerebellum. Highlighted and labeled genes are significant genes (FDR < 0.05) overlapped with MS-PRGenes

The study design and analysis pipeline are summarized in Fig. 1. Features used in the prioritization of MS-PRGenes and validations are shown in Table 1. In the current study, we applied a modified version of iRIGS [14] to prioritize MS-associated risk genes. We performed this analysis using the 200 risk lead SNPs reported in the previous GWAS of 14,802 MS cases and 26,703 control subjects [7]. Following the pipeline of iRIGS, we firstly identified 3,691 unique candidate risk genes – those genes were located within the 2-Mb region centered at each of the 200 MS-associated index SNPs. We subsequently added the genomic and epigenomic information into the algorithm. We integrated genomic and epigenomic data into the current analysis, including the distance from lead SNP locus to the transcription start site (TSS) of each candidate gene, Functional Annotation of the Mammalian Genome 5 (FANTOM5) data [23], genome-wide chromosome conformation capture (Hi-C) data [24, 25], and differentially expressed gene data of 21 post-mortem brain samples (11 MS cases and 10 control subjects) [17]. In addition, we integrated gene-level DNA methylation statistics using the data generated from 28 MS cases and 19 control subjects [8] (see Methods) into our modified Bayesian framework. Gene–gene relationships derived from the Gene Ontology (GO) network were adopted in the iRIGS framework (see Methods). Based on this integrative multi-omics analysis, we identified 163 unique MS-PRGenes with the highest posterior probability at each index SNP (Additional file 1: Table S1). Among them, prioritized genes *KMT2A* and *LITAF* possessed the highest posterior probability at four lead SNPs. Six genes (*CD48*, *CD86*, *CD9*, *MAF*, *MYC*, and *TNFAIP3*) had the highest posterior probability at three lead SNPs. There were 11 MS-PRGenes (Fig. 2A) with high posterior probabilities (≥ 0.75), including *MYC*, *LPIN1*, *MAF*, *ATXN1*, *JARID2*, *TNFAIP3*, *SATB1*, *GATA3*, *ELMO1*, *SGK1*, and *ZNF217*. Strikingly, 127 of 163 (77.91%) MS-PRGenes were not the closest genes to the corresponding index SNP loci.

**Table 1:** Summary of features used in the prioritization of MS-PRGs and validation

*Feature*	*Description*
*Bayesian framework (refined version of iRIGS) features*	
Genetic variants in MS	200 genetic variants from the currently largest MS GWAS with genome-wide significance
Functional Annotation of the Mammalian Genome 5 (FANTOM5)	Annotations of mammalian regulatory components, such as promoters, enhancers lncRNAs and miRNAs (provided by original iRIGS analysis)
Genome-scale chromosome conformation capture (Hi-C)	Brain Hi-C data including both short- and long-range interactions among genomic loci (provided by original iRIGS analysis)
Expression in MS brain tissue	Differentially expressed genes in MS brain tissue from 21 post-mortem brain samples (11 MS cases and 10 control subjects)
DNA methylation in MS brain tissue	Differentially methylated genes from epigenome-wide changes in DNA methylation levels of 28 MS cases and 19 control subjects
Gene–gene relationships	Gene interactions from Gene Ontology (GO) network (provided by original iRIGS analysis)
*Two-sample Mendelian randomization (2SMR) feature*	
Expression quantitative trait loci (eQTL)	Top cis-eQTL of 19 tissues based on tissue expression data from the Genotype-Tissue Expression (GTEx) portal
*Genetic features used in validation*	
Probability of loss of function (LoF) intolerant (pLI) scores	A high pLI score indicates the gene is more likely to be intolerant towards protein-truncating variant(s)
Evolutionary rate	The ratio of nonsynonymous over synonymous substitution rate (dN/dS)
Genes of human diseases	From OMIM and ClinVar databases
*Cell type features used in validation*	
Single-nuclei RNA-sequencing (snRNA-seq)	Cell type-specificity enrichment analysis using a snRNA-seq dataset from the brain tissue of 4 progressive MS patients and 5 non-neurological controls
*Drug features used in validation*	
MS drug targets	32 MS drug targets were collected from the DrugBank database, followed by the enrichment analysis
Connectivity Map (CMap) drug signatures	The co-expressed gene-set enrichment analysis (Cogena) R package was performed for the downregulated and upregulated 100 CMap gene sets

### Two-sample Mendelian randomization analysis

**Fig. 3:**
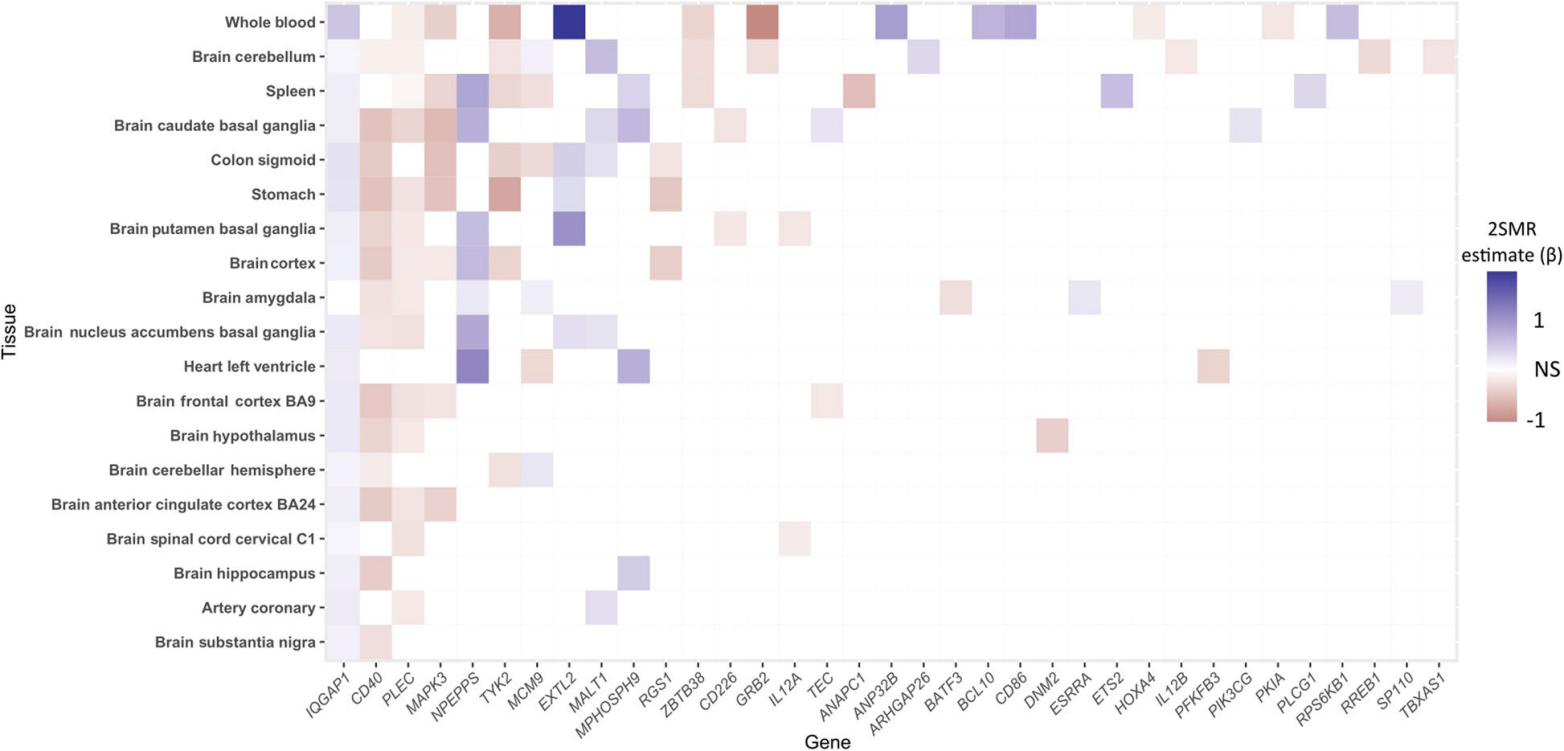
Heatmap of 2SMR analysis estimates of significant genes overlapped with MS-PRGenes in 19 tissues. Gene names are shown on the x-axis. Tissue names are shown in the y-axis ordered by the number of significant genes in each tissue. Red and blue are proportional to the effect size of each gene in each tissue

Two-sample Mendelian randomization (2SMR) is a cost-efficient method to estimate potential causal effects of gene expression-outcome relationships with SNP summary statistics from two independent datasets [26, 27]. To validate the gene list generated by modified iRIGS, we subsequently adopted the R package TwoSampleMR [20] to conduct 2SMR analyses on 3,691 candidate risk genes. We used eQTL as the genetic instruments generated from 19 relevant tissues [28] (Additional file 1: Tables S2-S21). The Benjamini-Hochberg (BH) procedure [29] was applied to the result of each tissue for multiple test corrections and to generate the respective false discovery rate (FDR). Of those genes that were significantly associated (FDR < 0.05) with MS in at least one tissue, we identified a total of 35 genes that were overlapped with 163 MS-PRGenes (Fig. 3, Additional file 1: Table S2). Among them, we found that the IQ Motif Containing GTPase Activating Protein 1 (*IQGAP1)* gene was consistently upregulated in MS cases among 18 of 19 tissues. The strongest association for *IQGAP1* was found in whole blood (Wald ratio estimate = 0.50, FDR = 3.39 × 10^–3^). We also identified CD40 molecule (*CD40)* and plectin (*PLEC)* genes that were downregulated in MS cases among 14 of 19 tissues. Moreover, we found the strongest association between upregulated *EXTL2* and MS in the whole blood (Wald ratio estimate = 1.83, FDR = 4.72 × 10^–9^). Overall, 2SMR analyses of whole blood, brain cerebellum, and spleen validated more MS-PRGenes than other tissues (Figs. 2B-2D, Additional file 1: Tables S3-S5).

### Gene features of MS-PRGenes

**Fig. 4:**
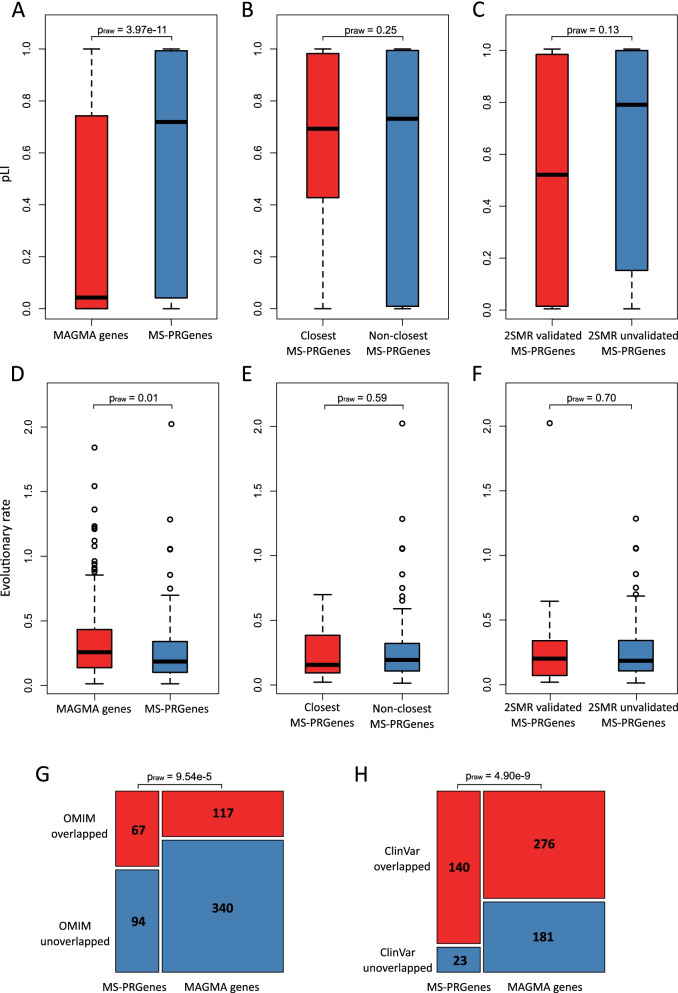
Exploration of the gene features of MS-PRGenes and MAGMA genes. (A) Comparing the boxplots of pLI scores between MS-PRGenes and MAGMA genes in a two-sided t-test. (B) Comparing the boxplots of pLI scores within the closest gene and non-closest gene groups of MS-PRGenes in a two-sided t-test. (C) Comparing the boxplots of pLI scores within the 2SMR validated gene and 2SMR unvalidated gene groups of MS-PRGenes in a two-sided t-test. (D) Comparing the boxplots of evolutionary rates between MS-PRGenes and MAGMA genes in a two-sided t-test. (E) Comparing the boxplots of evolutionary rates within the closest gene and non-closest gene groups of MS-PRGenes in a two-sided t-test. (F) Comparing the boxplots of evolutionary rates within the 2SMR validated gene and 2SMR unvalidated gene groups of MS-PRGenes in a two-sided t-test. (G) The proportional comparison of MS-PRGenes and MAGMA genes overlapped with disease genes parsed from OMIM in the chi-squared test. (H) The proportional comparison of MS-PRGenes and MAGMA genes overlapped with known genes with disease-associated variants parsed from ClinVar in the chi-squared test

To further validate the result of iRIGS, we conducted genetic feature analyses of MS-PRGenes. We compared the genetic features of MS-PRGenes with those of a gene set derived from the conventional Multi-marker Analysis of GenoMic Annotation (MAGMA, v1.07) tool [30]. By applying the MAGMA tool, we obtained the gene-level p-value related to MS. We then generated a gene set of 457 MAGMA genes, which we considered statistically significantly associated with MS after Bonferroni correction (p_Bonferroni_ < 0.05). To compare the genetic features, we firstly obtained the probability of being loss-of-function (LoF) intolerant (pLI) scores [31] for the 163 MS-PRGenes and the 457 MAGMA genes. Figure 4A shows that MS-PRGenes have significantly higher pLI scores than MAGMA genes (average pLI_MS-PRGenes_ = 0.57 (± 0.42), average pLI_MAGMA_ = 0.32 (± 0.40), p_raw_ = 3.97 × 10^–11^), indicating that MS-PRGenes are more likely to be deleterious. Sixty-one MS-PRGenes possessed a pLI score over 0.9, suggesting that those genes have a high probability of intolerance to loss-of-function variation. We subsequently compared the pLI scores within MS-PRGenes to investigate the difference between MS-PRGenes closer to index SNP and MS-PRGenes further away from index SNP. As shown in Fig. 4B, there is no significant difference between the pLI scores of 36 the MS-PRGenes closest to the index SNPs and the pLI scores of the 127 non-closest MS-PRGenes (p_raw_ = 0.25). Furthermore, we found no significant difference in the pLI scores between the 35 2SMR validated MS-PGRs and the other 127 MS-PRGenes (p_raw_ = 0.13) (Fig. 4C). These findings indicated that the pLI scores were consistent within MS-PRGenes.

Next, we explored the evolutionary selective pressure of both gene sets. The evolutionary selective pressure is defined as the ratio of the number of nonsynonymous substitutions per nonsynonymous site to the number of synonymous substitutions per synonymous site (dN/dS) [32]. It has been reported the disease genes tend to have lower evolutionary rates compared to non-disease genes [33]. After we parsed averaged evolutionary rates of 144 MS-PRGenes and 341 MAGMA genes from the previous study [33], we found MS-PRGenes had significantly lower averaged evolutionary rates than MAGMA genes (average dN/dS_MS-PRGenes_ = 0.26 (± 0.26), average dN/dS_MAGMA_ = 0.33 (± 0.27), p_raw_ = 0.01) (Fig. 4D). On the contrary, we found that the averaged evolutionary rates were consistent within groups of MS-PRGenes (Figs. 4E and 4F) (p_raw_ > 0.05).

Lastly, we compared the MS-PRGenes and MAGMA genes to explore if those genes are enriched in known human disease genes. We parsed 15,104 genes from Online Mendelian Inheritance in Man (OMIM) database with phenotype description [34]. We obtained 11,229 genes with disease variations from the ClinVar database [35]. As shown in Figs. 4G and 4H, the comparison with MAGMA genes indicated that more MS-PRGenes were overlapped with disease-related OMIM genes (p_raw_ = 9.54 × 10^–5^) and ClinVar genes (p_raw_ = 4.90 × 10^–9^).

### Single-cell RNA-sequencing context-specific enrichment analyses of MS-PRGenes

**Fig. 5:**
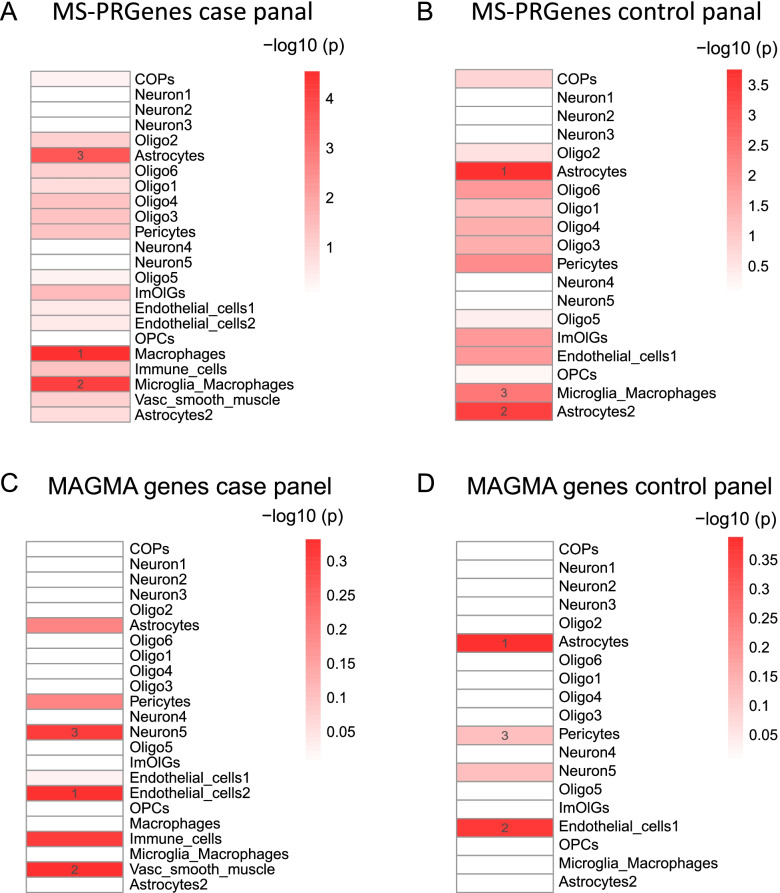
Single-cell context-specific enrichment analysis. (A, B) Single-cell context-specific enrichment analysis of MS-PRGenes in MS snRNA-seq case and control panels, respectively. (C, D) Single-cell context-specific enrichment analysis of MAGMA genes in MS snRNA-seq case and control panels, respectively

To understand the cell type and the context of how MS-PRGenes manifest their impact, we used one MS single-nuclei RNA-sequencing (snRNA-seq) dataset from the brain tissues of four progressive MS patients and five non-neurological controls [36]. By applying our previously developed tool of cell type-specificity enrichment analysis [21, 22, 37, 38], we identified that the MS-PRGenes and MAGMA genes varied from each other in MS case and control panels (Fig. 5). Interestingly, we identified MS-PRGenes were enriched in the top 3 cell types of Macrophages (p_raw_ = 2.86 × 10^–5^), Microglia_Macrophages (p_raw_ = 7.21 × 10^–5^), and Astrocytes (p_raw_ = 2.24 × 10^–4^) in the MS case panel (Fig. **5**A). Meanwhile, the top 3 enriched cell types in the control panel (Fig. 5B) were Astrocytes (p_raw_ = 1.76 × 10^–4^), Astrocytes2 (p_raw_ = 3.71 × 10^–4^), and Microglia_Macrophages (p_raw_ = 3.47 × 10^–3^). These findings indicated that MS-PRGenes tended to be highly expressed in activated macrophages and microglia macrophages. On the other hand, the enrichment of astrocytes remained stable in inflammatory and non-inflammatory tissues. Surprisingly, we failed to detect any significantly enriched cell types for MAGMA genes (Figs. 5C and D), suggesting that MAGMA genes might contain many false positive genes and miss some long-distance (> 50 kb) signals.

### Biological and drug pathways enriched for MS-PRGenes

**Fig. 6:**
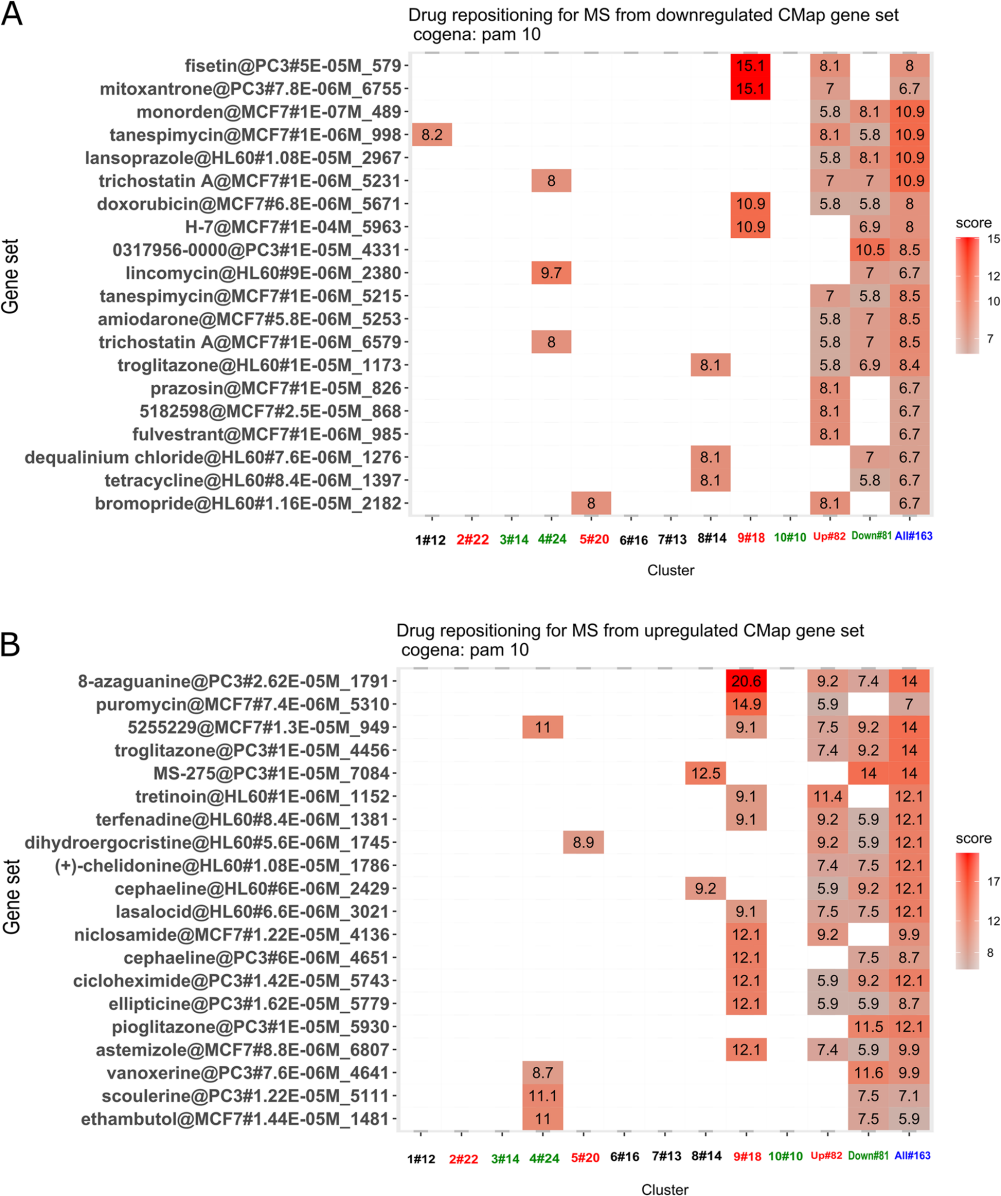
CMap drug signatures enriched in the MS-PRGenes depict potential drug repositioning strategies. (A, B) Drugs enriched for the MS-PRGenes are shown on the y-axis. Enrichment scores represent -log2 (false discovery rate), as reported by the *Cogena* R package. The color is proportional to the enrichment score. (A) Drugs listed on the y-axis show the enrichment in the drug signatures of downregulated 100 CMap gene set. (B) Drugs show the enrichment from the drug signatures of upregulated 100 CMap gene set

A total of 32 drug targets were identified for the FDA-approved drugs indicated for MS. Within the list of 163 MS-PRGenes, 3 MS drug targets were present: *HDAC1, IFNAR2,* and *RELA,* none of which had been reported in the independent multi-omics studies. The co-expressed gene-set enrichment analysis (Cogena) implemented in the Bioconductor R package was applied to examine gene functions by pathway and drug signature enrichment analyses (see Methods). The scores reported for the enriched pathways represent negative log2 FDR, as reported from the Cogena hypergeometric tests [39]. Biological pathway enrichments yielded a "toll-like receptor signaling pathway" as the top enriched KEGG pathway (score = 44.4 in all clusters). Other enriched KEGG pathways included "acute myeloid leukemia" (score = 33.1 in all clusters), "pathways in cancer" (score = 32.2 in all clusters), "T cell receptor signaling" (score = 28.3 in all clusters), "B cell receptor signaling" (score = 26.1 in all clusters), among other biological pathways (Additional file 2: Figure S1). Moreover, the MS-PRGenes were also enriched with drug signatures from both the downregulated and upregulated 100 Connectivity Map (CMap) gene sets. Top enriched drugs from the downregulated 100 CMap gene set included fisetin (score = 15.1 in cluster 9), mitoxantrone (score = 15.1 in cluster 9) and monorden (score = 10.9 in all clusters) (Fig. 6A). The drug signature enrichment analysis of upregulated 100 CMap gene sets in MS-PRGenes yielded enrichment of the drugs 8-azaguanine (score = 20.6 in cluster 9), the small molecule MS-275 (score = 14 in all clusters), and pioglitazone (score = 12.1 in all clusters), among other drugs (Fig. 6B).

## Discussion

In this study, we extended the Bayesian framework, iRIGS, and applied it to MS to prioritize 163 MS-PRGenes from 200 genetic loci that had been reported in the current largest MS GWAS. We validated 35 MS-PRGenes through parallel 2SMR analyses of 3,691 unique candidate genes on 19 tissues. MS-PRGenes demonstrated a higher disease relevance than other gene sets. We found MS-PRGenes had significantly higher pLI scores, lower averaged evolutionary rates, and higher overlap rates with MS-associated genes obtained from OMIM and ClinVar. Moreover, results of single-cell enrichment analyses showed that MS-PRGenes were enriched in macrophages and microglia among MS cases. Lastly, we explored potential drug repositioning strategies for MS by performing drug signature enrichment analysis of the MS-PRGenes. Drug signature enrichments depicted several drugs associated with MS mechanisms. We specifically discussed the therapeutic potentials of fisetin, monorden, 8-azaguanine, the small molecule MS-275, and pioglitazone in MS.

MS-PRGenes were aligned with known disease risk genes. The strongest association of 2SMR was found between upregulated *EXTL2* and MS in whole blood. The genetic role of *EXTL2* has been validated in an animal study, which reported that *EXTL2* deficiency was associated with exacerbated axonal damage and myelin disruption [40]. Several MS-PRGenes with high posterior probabilities (> 0.75), such as *MYC*, *ATXN1*, and *GATA3*, have also been reported in recent MS studies [41–43].

MS-PRGenes are more deleterious and disease-related than the genes identified by the conventional MAGMA tool. We found that MR-PRGenes had significantly higher pLI scores than MAGMA genes (Fig. 4A), and 61 of them possessed pLI scores greater than 0.9. Of interest, the *IQGAP1* gene possessed a pLI score over 0.99. We observed consistent associations that the *IQGAP1* gene was overexpressed in MS cases in 2SMR analyses in 18 of 19 tissues (Fig. 3). This result aligns with a previous study, which shows that individuals with a homozygous MS risk allele have a significantly higher expression of *IQGAP1* compared to controls [44]. However, it is still unclear how higher *IQGAP1* expression could increase the risk of MS. A study has indicated that *IQGAP1* may play an important role among cytoskeleton-mediated processes in immune cells [45]. It has been hypothesized that increasing *IQGAP1* expression might lead to abnormal leukocyte cell migration and thereby contribute to MS disease [44]. Moreover, tumor necrosis factor alpha-induced protein 3 (*TNFAIP3*) gene, identified through iRIGS with a posterior probability over 0.75, also possessed a pLI score greater than 0.99. A previous study shows that *TNFAIP3* expression level is diminished in monocytes and CD4 + T cells of MS patients, suggesting *TNFAIP3* plays a role in anti-inflammatory pathways, which might contribute to MS risk [46]. Furthermore, the averaged evolutionary rates of MS-PRGenes were significantly lower than those of MAGMA genes (Fig. 4D). The result suggested that MS-PRGenes might have under stronger selective pressure than MAGMA genes. This is consistent with a previous study [33], which has reported that disease genes tended to have lower averaged evolutionary rates than non-disease genes. We also found MS-PRGenes contained more known disease genes than MAGMA genes (Figs. 4G and H**)**, adding more confidence to the result of iRIGS.

Our snRNA-seq context-specific enrichment analyses showed that MS-PRGenes tended to be more activated in immune function-related cells (macrophage and microglia) in the disease state. This is consistent with the report by Mu et al. that human immune traits, including MS, have disease context-dependent regulatory factors [47]. On the other hand, our finding aligned with the fact that MS is a chronic neurological disease with proinflammatory dysregulation in the central nervous system [48] (Fig. 5). The complex roles of microglia and macrophages on MS pathology have been widely reported [49]. The activation of microglia and macrophages in the early phase of the disease is associated with increased secretion of proinflammatory cytokines, such as TNF-α, leading to demyelination and axonal loss [50]. While in the later stage, the activation of microglia and macrophages are associated with disease alleviation by secreting neuroprotective molecules and promoting remyelination [51]. However, the conventional MAGMA genes failed to capture this signal. Among MS-PRGenes, studies have shown the *CD40* gene broadly expressed in macrophages and activated microglia, while the expression level of *CD40* is low during resting state [52, 53]. In our 2SMR analyses (Fig. 3), *CD40* was lowly expressed (Wald ratio estimate < 0) in 12 of 13 brain tissues among MS cases. On the other hand, *CD86*, another MS-PRGene, has been reported as co-expressed with *CD40*. Both genes belong to the M1-type maker (classically activated macrophage) of macrophages, activated microglia, and myelin-loaded macrophages in MS lesions [52]. Based on the result of 2SMR analyses, we found no significant association between *CD86* expression and MS in all brain tissues, but *CD86* was upregulated among whole blood (Wald ration estimate > 0). This discrepancy might indicate a tissue-specific expression pattern. A recent single-cell transcriptome study has reported diverse transcriptional changes of blood and cerebrospinal fluid leukocytes [54].

Pathway enrichment analysis of the MS-PRGenes could yield relevant functions that have implications for disease mechanisms (Additional file 2: Figure S1). The top enriched KEGG pathway for the MS-PRGenes was "toll-like receptor signaling pathway." Toll-like receptors are receptors of the innate immune system that recognize invading pathogens. Toll-like receptors may play a role in the pathogenesis of autoimmune diseases, like MS, by fighting suspected infections and activating autoimmune reactions [55]. Interestingly, although we integrated multi-omics data from brain tissue samples, we also highlight KEGG pathways enriched for immune-related pathways, such as "T cell receptor signaling pathway," "B cell receptor signaling pathway," and "chemokine signaling pathways." This suggests that there is infiltration of peripheral immune cells in the MS brain samples. Single-cell profiling of neuroinflammatory states has shown that infiltration of peripheral immune cells indeed occurs during neuroinflammatory pathologies [56]. Several cancer-related KEGG pathways were also enriched in our MS-PRGenes, such as "acute myeloid leukemia," "pathways in cancer," and "chronic myeloid leukemia." The link between MS and cancer has previously been investigated [57].

Drug targets of MS FDA-approved drugs were present in the MS-PRGenes. Drug targets of MS medications present in the list of MS-PRGenes were *HDAC1, IFNAR2,* and *RELA*. These genes have not been reported by MS GWAS, transcriptomic or epigenetic study. *HDAC1*, a gene that encodes for histone deacetylase, is a drug target of the MS medication fingolimod, which may play a role in epigenetic mechanisms of MS. In the mechanism of action of fingolimod, *HDAC1* is inhibited, thereby allowing specific histone acetylation to occur. This drug target has also been identified as central by network-based analyses of previous MS GWAS [58]. *IFNAR2* is a target of the MS medication interferon beta-1, and polymorphisms of *IFNAR2* have previously been implicated in the susceptibility of MS [59]. *RELA* is a drug target of the medication dimethyl fumarate. The *RELA* gene encodes an important mediator of the NF-kB signaling pathway, which plays a critical role in cellular immune responses. It has been demonstrated that the NF-kB signaling pathway is dysregulated in MS patients, and that *RELA* is particularly increased during progressive phases of the disease [60].

Enrichment of downregulated and upregulated 100 CMap gene sets was also observed in drug signature enrichment analyses of MS-PRGenes. The top enriched drugs for downregulated 100 CMap signatures included fisetin, mitoxantrone, and monorden (Fig. 6A). Mitoxantrone, in particular, is a chemotherapeutic medication that has been used to treat progressive forms of MS. Mitoxantrone is an efficacious and low-cost therapy for MS; however, it may pose safety concerns with a risk profile of potential adverse effects [61]. Fisetin, another enriched drug, is a dietary compound that may possess neuroprotective qualities. Recent preclinical models have demonstrated that it reduces the progression of several neurological disorders, including MS [62]. Monorden, also known as radicicol, is an experimental small molecule and antifungal antibiotic. It may also possess neuroprotective effects, as studies of monorden with animal models have shown that monorden prevented cell death and diminished tumor necrosis factor (TNF) in neuron-glia cultures [63]. This suggests that monorden may be a potential therapy for diminishing neuron cell death in neurodegenerative diseases like MS. The top enriched drugs with upregulated 100 CMap signatures included 8-azaguanine, the small molecule MS-275, and pioglitazone (Fig. 6B). The small molecule 8-azaguanine has been previously used to treat acute myeloid leukemia, as it was initially developed as an antineoplastic agent. However, in recent years, 8-azaguanine has been identified as an immunomodulatory agent for its cytotoxic effects on natural killer cells [64]. Immunomodulatory actions of 8-azaguanine may be considered for MS treatment. Pioglitazone is a medication which has been used to treat diabetes. The potential of pioglitazone as a treatment for MS has been investigated in a mouse model of experimental autoimmune encephalomyelitis (EAE). Oral administration of pioglitazone reduced white matter loss in the spinal cord of mice, and it was concluded that pioglitazone may decrease myelin damage and inflammation in MS [65]. Finally, MS-275 is another experimental compound that inhibits histone deacetylases. Because *HDAC1* is already a target of FDA-approved MS medications, the compound MS-275 also shows promise for MS treatment.

In the current study, we modified the iRIGS algorithm to highlight MS-PRGenes by integrating GWAS summary statistics, gene expression, DNA methylation, FANTOM5, and brain genome-scale chromosome conformation capture (Hi-C) data. We think the resulting MS-PRGenes are highly confident, as the algorithm harmonized accumulating multi-omics signals that perform complex biological functions collaboratively. However, the results should be interpreted with caution. First, we only selected the candidate risk gene with the highest sampling frequency at each locus as the MS-PRGene. Although the current algorithm can rank all candidate risk genes by their sampling frequency, iRIGS cannot detect how many risk genes that are truly associated with MS. Besides, the brain-specific Hi-C data used in the current study were generated from the cortical and subcortical plate of the fetal brain [25]. As we discussed previously, the risk genes expression of immune traits, including MS, may be associated with disease context-dependent regulation [47]. Thus, we believe the MS risk genes prediction accuracy will be further increased if more disease context-related epigenomic and Hi-C data are incorporated into the algorithm.

## Conclusions

To conclude, we modified the Bayesian framework of iRIGS by integrating disease-specific multi-omics data of MS. We prioritized 163 genes for MS risk (MS-PRGenes). We further conducted a series of analyses, including eQTL analyses, 2SMR, single-cell RNA-seq context-specific enrichment analyses, and gene features exploration, to validate the MS-PRGenes and gene-based MAGMA genes. Lastly, we performed CMap drug signature enrichment analyses and identified several potential drugs that could be repurposed for MS treatment. Overall, our MS-PRGenes optimize the information from multi-omics data and demonstrate better performance than conventional methods, which could serve as a high-confidence MS risk gene set and benchmark.

## Methods

### GWAS summary statistics

MS GWAS summary statistics were retrieved from a comprehensive genetic association study conducted by International Multiple Sclerosis Genetics Consortium (IMSGC) [7]. The summary statistics collected from the IMSGC website (accessed on 3/29/2019) corresponded to the discovery set of this study, which included 8.86 million SNPs from 14,802 MS cases and 26,703 controls. In addition, they conducted rigorous quality checks for all data sets, and the genotype data were imputed based on the 1000 Genomes Project reference panel [66]. In total, 233 genetic variants were reported, while 200 autosomal susceptibility variants outside the major histocompatibility complex were used in the following analysis.

### Gene expression data

We obtained the gene expression data from a recent study which profiled expression data of post-mortem brain tissue of 10 MS cases and 11 control subjects (Gene Expression Omnibus (GEO) accession ID: GSE111972 on 11/15/2020) [17]. The original study generated expression data from microglia samples of white matter and grey matter using Illumina NextSeq500 SR75 kits. Here, we analyzed the samples derived from white matter. Raw read counts were normalized through R package *DESeq2* [67], and then *DESeq2* was used to obtain differentially expressed genes between white matter samples of 10 MS cases and 11 control subjects. Raw p-values generated from the differentially expressed gene analysis were used for the multi-omics analysis of risk gene prioritization.

### Methylation data

We approached the methylation data from an epigenome-wide difference study of 28 MS cases and 19 control subjects (GEO accession ID: GSE40360 on 2/1/2021) [8]. Post-mortem brain frontal lobe specimens were dissected, and global DNA methylation levels were assessed using Illumina Infinium HumanMethylation450 BeadChip. Firstly, we used the *limma* package to obtain the association values for each CpG probe [68]. Methylation analyses typically perform single-CpG differentially methylated probes analysis and differentially methylated regions analysis. However, neither of these methods was suitable for obtaining gene-level methylation scores. Here, we used the promoter region CpGs as the surrogate for gene-level methylation, as they have been reported regulating the gene expression. Specifically, we adapted the CpGs annotated in the transcription start site (TSS) of genes. This information was obtained from the GPL13534 annotations file for HumanMethylation450 BeadChip. To generate gene-level methylation scores, we firstly calculated a Z score for each CpG probe based on respective raw p-values and the log fold changes yielded by *limma*. Next, we used Stouffer's Z score method [69] to combine p-values, following the formulas:

$${Z}_{CpG}=Sign\left({log}_{2}FC\right)\times {\varphi }^{-1}(\frac{p}{2})$$

$${Z}_{M}=\frac{\sum_{i=1}^{k}{Z}_{{CpG}_{i}}}{\sqrt{k}}$$

where ZCpG is the z-score pertaining to a CpG probe, Sign (log2FC) is the sign (+ or -) of fold change, p is the p-value of CpG probe, ZM is the methylation score for a particular gene, and k is the number of CpGs annotated for a gene. Lastly, we reversed gene-level z values to gene-level p-values for each mapped gene. The gene-level p-values were used in following risk gene prioritization.

### Multi-omics data integration and risk gene prioritization

We adopted a newly developed Bayesian algorithm, integrative risk gene selector (iRIGS), to prioritize MS-associated genes from the result of the largest MS GWAS and additional multi-omics data [14]. The detailed methods can be found in the original publication [14]. In brief, all genes within two Mb regions centered at each index SNP (200 index SNPs in the current study) were identified as candidate risk genes. The algorithm aims to identify a set of candidate risk genes with maximized posterior probability considering the integrated multi-omics evidence and a predefined gene–gene network.

To tackle the computational infeasibility from calculating all gene combinations, iRIGS applied a Gibbs sampling algorithm [70] to sample only one gene at a single locus per iteration, assuming risk genes at other loci have been selected. At each iteration, the sampling algorithm was treated as a Bayesian model selection question that each candidate gene in a locus was seen as a model. The prior odds of each model were derived from the predefined network using the walk with restart algorithm. The original publication provided the predefined network that the weighted network was calculated using information from the Gene Ontology database [71]. The iRIGS uses integrated multi-omics evidence as the surrogate for the Bayes Factor in the model. Specifically, the algorithm employed the Mahalanobis decorrelation transformation [72] on FANTOM5, distance to TSS, and Hi-C data. First, these data were transformed into independent and identically distributed random variables, each following a univariate standard Gaussian distribution. The p-value of each of the above features was subsequently calculated. Then, the p-values from differentially expressed genes analysis data and gene-level methylation data were concatenated to generate a p-value matrix. Finally, the iRIGS applies Fisher's product method to integrate all p-values of each gene, and the products were used as the surrogate for the Bayes Factor in the model.

With the prior odds and Bayes Factor information, the iRIGS iteratively sampled one candidate gene at a time. The sampling frequency of all candidate genes was calculated after each round of the sampling process on all risk SNPs. The process was repeated until the sampling frequency reaches a stationary distribution, defined as the sum of squares of frequency difference of selected genes was smaller than a preset threshold (0.01 was used in the current study).

In the current study, we adopted a modified version of iRGS on 200 risk SNPs identified from GWAS of MS mentioned in the previous method (Fig. 1) [7]. Following their method, we integrated multi-omics data into the algorithm, including FANTOM5 data, which provide critical links between regulatory elements and the genes that they regulated [23]. Then, the distances between the index SNP to TSS of candidate genes were calculated. Next, brain genome-scale chromosome conformation capture (Hi-C) data were incorporated, which provide global views of both short- and long-range interactions among genomic loci [24, 25]. FANTOM5 data and Hi-C data were available in the original iRIGS method. In addition, differentially expressed gene data generated from 21 post-mortem brain samples (11 MS cases and 10 control subjects) [17] were included as well. Compared to the original iRIGS method, we additionally integrated differentially methylated gene data generated from 47 post-mortem brain samples (28 MS cases and 19 control subjects) [8]. All multi-omics data were integrated using the method mentioned above. Finally, we defined the genes with the highest sampling frequency at each locus as the MS-PRGenes.

### Comparison with conventional GWAS-based gene-level p-value

To compare the MS-PRGenes with another gene set, we applied a Multi-marker Analysis of GenoMic Annotation (MAGMA, v1.07) [30]. The MAGMA tool calculated the gene-level p-values, from which we generated a conventional MAGMA gene set. Briefly, SNPs located within 50 kb upstream and 35 kb downstream of the gene body were mapped to each gene. We computed the gene-level p-values based on mean χ^2^ statistics for these SNPs. The effects of the gene length, SNP density, and local linkage disequilibrium (LD) structure were considered in MAGMA analysis. The 1000 Genome Project Phase 3 European population was used as the reference panel. We performed Bonferroni correction on MAGMA gene-level p-values. Genes with p_Bonferroni_ < 0.05 were defined as MAGMA genes.

### Two-sample Mendelian randomization analysis

Mendelian randomization (MR) is a statistical method for evaluating causality between an exposure and an outcome using genetic variants as instrumental variables. 2SMR method was subsequently developed to overcome the challenge that individual-level data is not always available [73]. With the growing number of summary statistic data available in public, 2SMR analysis provides a cost-efficient way to explore the potential causal effects of gene expression on MS [26, 27]. To validate the result of MS-PRGenes, we conducted the paralleled 2SMR analyses using the same candidate risk genes for the iRIGS model among various tissues.

Following the R package *TwoSampleMR* [20], we firstly inquired Genotype-Tissue Expression (GTEx v8) Portal [28]. We obtained the top cis-expression qualitative trait locus (cis-eQTL) (p_raw_ < 1 × 10^–4^) for all candidate risk genes, which were identified in the previous step as genetic instrumental variables. We obtained top cis-eQTL of 19 tissues, including spleen, whole blood, all brain tissues (13 tissues), as well as stomach, heart left ventricle, artery coronary, and colon sigmoid. Considering that eQTL may be associated with disease SNP due to linkage disequilibrium (LD) patterns, we performed LD clumping on each set of SNPs. We used a function provided by *TwoSampleMR* to remove all SNPs present in the 1000 Genomes European population with r^2^ > 0.001 and within 10 Mb of the top SNPs [20]. With remaining SNPs, we extracted and harmonized matched summarized data from outcome GWAS [7]. We subsequently conducted 2SMR on harmonized data for each tissue using the Wald ratio method if one independent SNP remained for the candidate risk gene. Otherwise, we used the inverse-variance weighted method if multiple SNPs were mapped to the candidate risk gene after LD clumping. Benjamin-Hochberg (BH) procedure [29] was applied for multiple test correction with a false discovery rate (FDR) of 0.05 as the threshold.

### Single-cell RNA-sequencing context-specific enrichment analysis

We obtained one MS snRNA-seq dataset (GEO accession ID: GSE118257 on 11/22/2019) of 20 brain samples from 4 progressive MS patients (11,208 cells) and 5 non-neurological controls (6,591 cells) [36]. We adapted their cell type annotation and implemented our t-statistics-based method [37, 38] to construct the disease context-specific panels for MS cells and health control cells. Specifically, we filtered out genes with more than a 95% read count equal to 0, leaving 5,659 genes in the case panel (23 cell types) and 7,657 genes in the control panel (19 cell types). Lastly, we applied our previous cell type-specific enrichment analysis [21] to detect the cell type-specificity of candidate genes.

### Pathway and drug signature enrichment analyses

Drug targets were collected for FDA-approved drugs indicated for MS from querying the DrugBank database [74]. A drug target query was performed for the MS-PRGenes, based on the collection of MS drug targets. We also performed pathway and drug signature enrichment analyses by using Cogena Bioconductor R Package [39]. Cogena is a framework that calculates the co-expression of input genes to determine gene expression signatures and create clusters associated with the disease mechanisms. Cogena uses hypergeometric tests to perform gene set enrichment analysis of biological pathways or curated drug signature gene sets. The input for Cogena is a gene expression matrix for the prioritized genes. Here, we constructed a gene expression matrix with the MS-PRGenes identified from the MS multi-omics data. Then, we used this matrix as the input for Cogena. The gene expression matrix included 10 MS cases and 11 controls and the corresponding RNA-seq expression values from brain tissue expression for each MS-PRGene. The default parameters were used to perform pathway and drug signature enrichment analyses: ten clusters, two cores, hierarchical and pam methods for clustering methods, and correlation for distance metric. We used three curated gene sets from the *Cogena* R package to perform these analyses: the KEGG gene set, the connectivity map (CMap) gene set for the top 100 downregulated genes per drug, and the CMap gene sets for the top 100 upregulated genes per drug.

## Supplementary Information

**Additional file 1.**

**Additional file 2. **

## Data Availability

The datasets analyzed during the current study are available. The summarized GWAS statistics are available at https://science.sciencemag.org/content/365/6460/eaav7188/tab-figures-data. Gene expression data can be downloaded from Gene Expression Omnibus (GEO) under the accession ID: GSE111972. Methylation data can be downloaded from GEO under the accession ID: GSE40360. Cis-expression qualitative trait locus data can be obtained from The Genotype-Tissue Expression (GTEx) website (https://gtexportal.org/home/). The source code of the current study and the supporting files are provided at Github (https://github.com/bsml320/MS-multioimcs_Bayesian_prioritization).

## Abbreviations

2SMR: Two-sample Mendelian randomization
eQTL: Expression quantitative trait loci
FANTOM 5: Functional Annotation of Mammalian Genomes 5
GEO: Gene Expression Omnibus
GWAS: Genome-wide association studies
iRIGS: Integrative Risk Gene Selector
MAGMA: Multi-marker Analysis of GenoMic Annotation
MS: Multiple sclerosis
MS-PRGenes: MS-associated prioritized risk genes
OMIM: Online Mendelian Inheritance in Man
pLI: Probability of being Loss-of-function intolerant
SNP: Single nucleotide polymorphism
TSS: Transcription start site
