# Supplementary figures and images for: Prioritization of risk genes in multiple sclerosis by a refined Bayesian framework followed by tissue-specificity and cell type feature assessment
Source: BMC Genomics. 2022 May 11;23(Suppl 4):362. doi: 10.1186/s12864-022-08580-y (PMC9092676; doi:10.1186/s12864-022-08580-y)

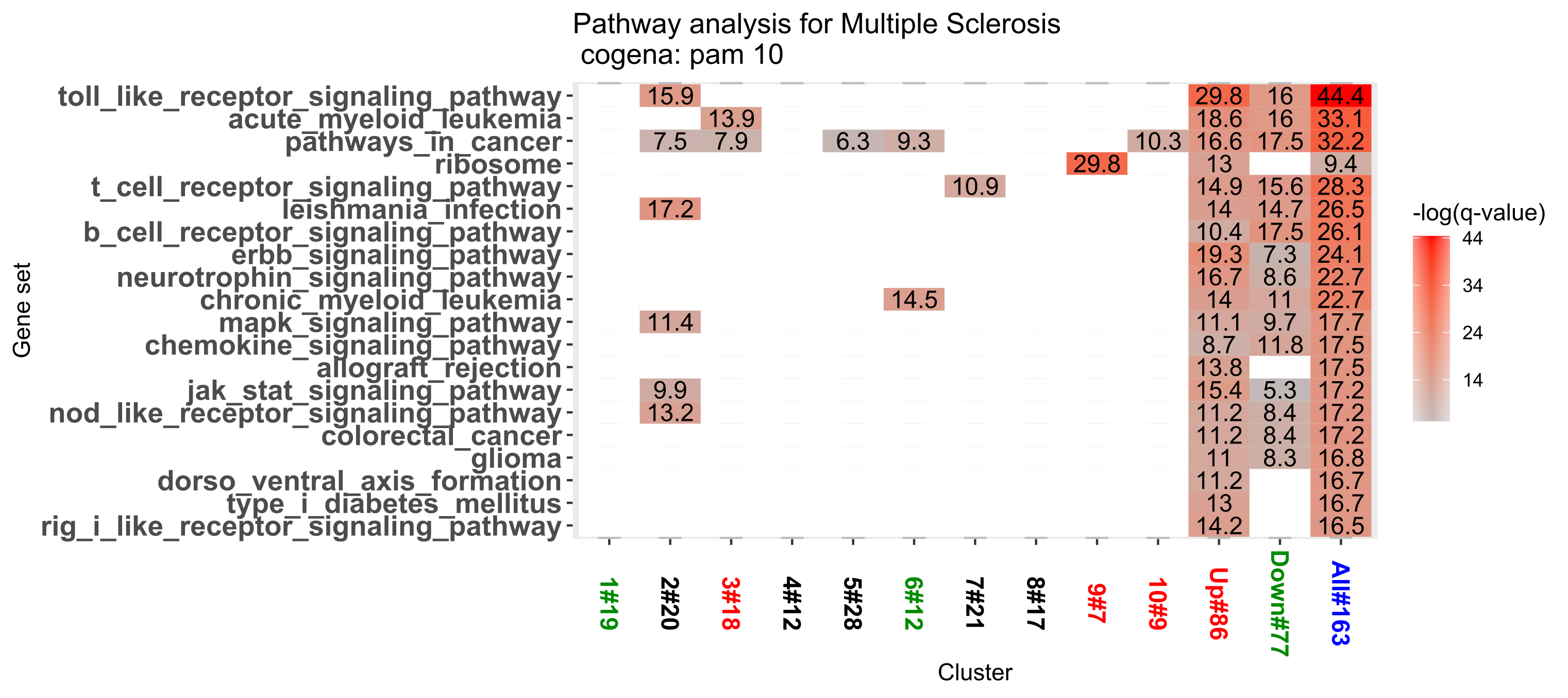

Supplement: Supplementary file 2 — Additional file 2. [file 12864_2022_8580_MOESM2_ESM.png]
